# Frustration of crystallisation by a liquid–crystal phase

**DOI:** 10.1038/srep42439

**Published:** 2017-02-17

**Authors:** Christopher D. Syme, Joanna Mosses, Mario González-Jiménez, Olga Shebanova, Finlay Walton, Klaas Wynne

**Affiliations:** 1 School of Chemistry, WestCHEM, University of Glasgow, UK; 2 Diamond Light Source, Harwell Science and Innovation Campus, Oxfordshire, UK

## Abstract

Frustration of crystallisation by locally favoured structures is critically important in linking the phenomena of supercooling, glass formation, and liquid-liquid transitions. Here we show that the putative liquid-liquid transition in *n*-butanol is in fact caused by geometric frustration associated with an isotropic to rippled lamellar liquid-crystal transition. Liquid-crystal phases are generally regarded as being “in between” the liquid and the crystalline state. In contrast, the liquid-crystal phase in supercooled *n*-butanol is found to inhibit transformation to the crystal. The observed frustrated phase is a template for similar ordering in other liquids and likely to play an important role in supercooling and liquid-liquid transitions in many other molecular liquids.

Cooling a liquid should cause it to crystallise. It has been known for more than a century^1^ that this process may occur in a number of steps where the degree of order gradually increases. Thus on cooling, an isotropic molecular liquid may form a nematic liquid-crystal (LC) phase in which the molecules have a degree of orientational ordering^2^. On further cooling, smectic LC phases may form that have partial translational order as well. Supercooling of the isotropic liquid below the liquid–LC transition is generally unimportant because the interfacial energy—the energy required to establish an interface between two phases—is very low^3^. This is caused by the significant structural similarity between the phases, which only differ in their orientational ordering.

Some supercooled liquids exhibit liquid–liquid transitions between two apparently amorphous phases. Although polyamorphic liquid–liquid transitions have been observed in a number of strongly interacting atomic liquids^4^,^5^,^6^, in molecular liquids a liquid–liquid transition has only been described in triphenyl phosphite, diphenyl-bithiophene-based polycatenar compounds, and *n*-butanol^7^,^8^,^9^,^10^,^11^,^12^. The existence of liquid–liquid transitions in molecular liquids has been highly controversial and has been dismissed as “aborted crystallisation”^13^,^14^,^15^. Here we will show that supercooled *n*-butanol does not undergo an amorphous-to-amorphous liquid-liquid transition but in fact undergoes a transition from an isotropic liquid to an LC phase. A liquid–LC transition is not only remarkable for such a small molecule but it is also unique in that it is not intermediate between the liquid and the crystal but instead geometrically frustrates the formation of the latter.

## Results

*N*-butanol has a melting temperature of *T*_*m*_ = 183 K and is a glass former with an observed glass-transition temperature of *T*_*g*_ = 118 K^16^,^17^. On quenching *n*-butanol with a rate of 20 K/min to a temperature below *T*_*m*_, we observe a transition in which the original liquid transforms into a chemically identical but physically different liquid. This transition is observed in our experiments at quenching temperatures between 120 and 150 K consistent with previous observations, which assigned this to a polyamorphic liquid-liquid transition^8^,^17^.

**Figure 1:**
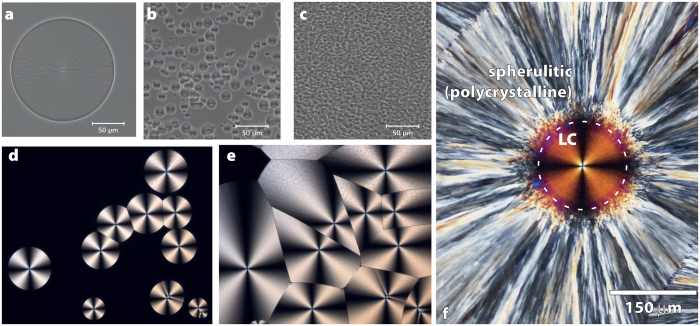
Phase-contrast and polarisation microscopy of a new phase nucleating in n-butanol. The top panels show phase-contrast images taken at the approximate halfway point in the transformation demonstrating nucleation. (**a**) 140 K (56 minutes), (**b**) 130 K (80 minutes), (**c**) 128 K (83 minutes). The lower panels show polarisation microscopy images exhibiting Maltese cross patterns consistent with liquid-crystal (LC) ordering. Quenched to 136 K and held for 2 hours (**d**) and 4 hours (**e**) showing nucleation, growth, and complete conversion to the LC phase. (**f**) The rightmost panel shows a polarisation microscopy image of a droplet (outline indicated by a dashed circle) surrounded by polycrystalline spherulitic growth after a cold-crystallisation cycle, showing that the LC droplets resist crystallisation.

*N*-butanol was investigated using phase-contrast and polarisation microscopy. Phase-contrast microscopy is sensitive to changes in sample density while polarisation microscopy is sensitive to molecular alignment^9^. As can be seen in Fig. 1, at quenching temperatures below 150 K, the new phase nucleates as spherical droplets that continue to grow until all of the original liquid is converted to the new phase. As the quenching temperature is lowered, the number of nucleation sites increases rapidly as expected for a homogeneous nucleation process. However, the rate of growth of the nucleated droplets decreases with temperature as the viscosity increases as the glass transition is approached. On repeating the experiments with the same sample, it is seen that the droplets nucleate in random positions demonstrating that heterogeneous nucleation does not play a major role.

Figure 1 also shows polarisation microscopy data taken during the transformation to the new phase. It shows high-contrast Maltese cross patterns inside the droplets characteristic of a highly ordered LC phase with a director pointing away from the centre of the droplet. This Maltese cross pattern is observed with the same contrast at all quenching temperatures in the range 128 to 145 K (see Figure S6). Thus, instead the putative liquid-liquid transition appears to be a transition from an isotropic liquid to a highly viscous LC phase while a crystalline phase is not observed to form on experimentally accessible timescales (hours).

Raman spectroscopy and mapping experiments were carried out that require a relatively long acquisition time. Therefore, a means had to be found to arrest the dynamics during these experiments. Hence, most of the experiments discussed below were carried out by rapid quenching (20 K/min) to 140 K to nucleate an approximately 50-μm diameter droplet of the LC in about 20 minutes, followed by either: rapid quenching to 110 K to arrest dynamics or: a cold-crystallisation cycle consisting of an increase of the temperature to 173 K followed by a rapid quench to 110 K. The latter causes the untransformed liquid to crystallise with spherulitic growth of crystals away from the droplet interface (see Fig. 1f and Figure S7 for the normal polycrystalline phase). These crystals do not penetrate the droplet. Additional changes are seen inside the droplet using polarisation microscopy with the Maltese cross pattern becoming weaker and the appearance of small grains.

**Figure 2:**
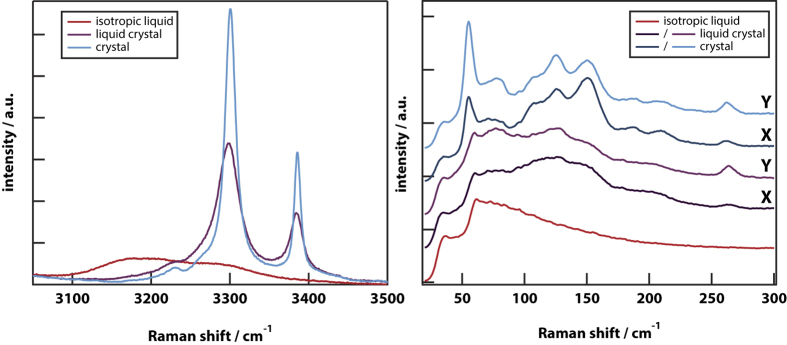
Raman spectra demonstrating the degree of ordering in the LC droplet before and after cold crystallisation. The Raman spectra of *n*-butanol taken before transformation (isotropic liquid, spectrum taken at 140 K), after the phase transition at 140 K (LC, spectrum taken at 110 K), and after a subsequent cold-crystallisation cycle (crystal, spectrum taken at 110 K). Spectra in the droplet (before and after cold crystallisation) were taken displaced along X and Y from the droplet centre. (**left**) Raman spectra in the OH-stretch region measured displaced along the Y-axis only. (**right**) Raman spectra in the low-frequency (phonon) region.

**Figure 3:**
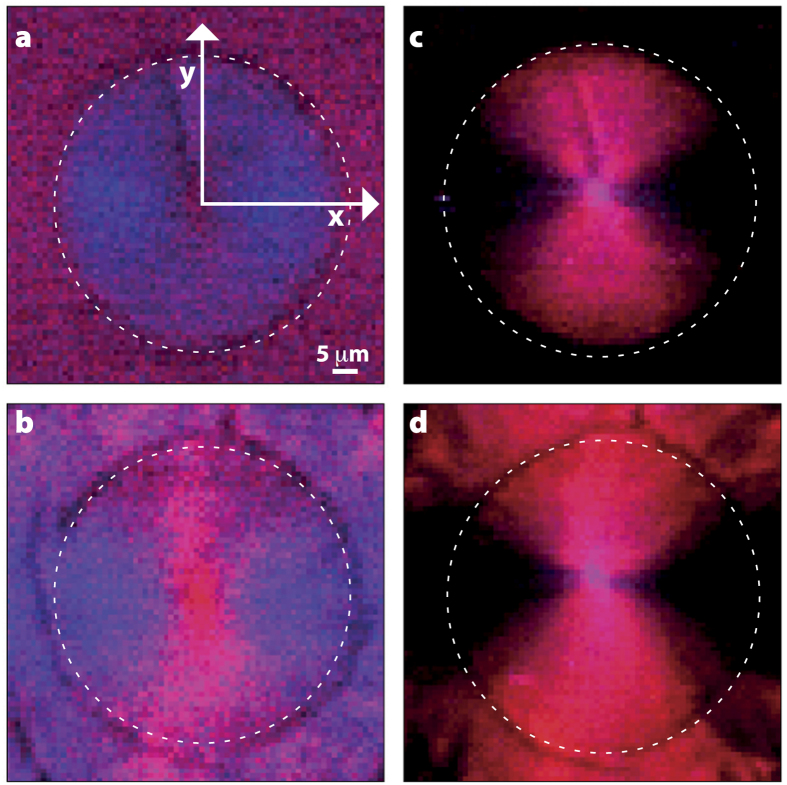
Raman maps of a droplet before and after cold crystallisation showing the degree of molecular ordering. Panels (a) and (b) show Raman maps of the low-frequency phonon region (the 55–60 cm^−1^ range shown in red and the 115–130 cm^−1^ range shown in blue) while panel (c) and (d) show maps of the OH-stretch region (the 3285–3305 cm^−1^ range shown in red and the 3370–3390 cm^−1^ region shown in blue). See Materials and Methods and S2 for details. Panels (a) and (c) are of a droplet (outline shown as a white circle) of LC grown by quenching to 140 K while panels (b) and (d) are the same sample after undergoing a cold-crystallisation cycle. All the data were taken at 110 K.

Raman spectra (Fig. 2) and Raman spectral maps (Fig. 3) were taken of isotropic liquid, LC droplets, and the same droplets after a cold-crystallisation cycle. On cooling *n*-butanol from room temperature, the OH-stretch band red shifts and narrows indicative of the formation of strong hydrogen bonds and local structure^13^,^18^. The liquid–LC transition causes the Raman spectrum to change significantly: it becomes more like the crystalline phase but is distinct. Thus, the OH-stretch bands in the crystal and LC are blue shifted and much narrower than in isotropic liquid. However, these bands in the LC are broader than the crystal indicative of greater disorder. A cold-crystallisation cycle only causes minor narrowing of the OH-stretch bands towards the width seen in the crystal. The OH-stretch spectrum around 3100 cm^−1^ can be used to estimate the degree of contamination of the LC with isotropic liquid (see S1). This contamination is found to be less than 2.8%, demonstrating full conversion to a new phase.

The intensity of the OH-stretch Raman band is highly anisotropic within the LC droplet as can be seen from the Raman map in Fig. 3. The OH-stretch band anisotropy (see S2) is ~5, that is, the Raman intensity is 5× larger in locations displaced from the droplet centre along the y-axis compared to the x-axis. Theoretical modelling (see S3) predicts a maximum Raman anisotropy for the crystalline phase of 7.6 while Raman microscopy experiments on polycrystalline *n*-butanol gives a maximum anisotropy of ~5–10. Thus, the LC phase in the droplet appears to be highly ordered. After a cold crystallisation cycle, it is seen that the Raman anisotropy within the droplet remains at ~5 while that of the spherulitic crystal growth outside the droplet is also about 5.

While the Raman spectral changes in the OH-stretch region are major, those in the low-frequency phonon region are more subtle (see Fig. 2). In the isotropic liquid, there are broad featureless bands from ~50–200 cm^−1^ corresponding to librations of butanol at low frequency and hydrogen-bond modes at higher frequency^19^. When the LC is nucleated at 140 K and the Raman spectrum obtained directly at 110 K, the low-frequency Raman spectrum observed is very similar to that of isotropic liquid with the same librational and hydrogen-bonding modes^20^,^21^. Additional structure is seen in the 60–300 cm^−1^ range indicating a degree of order. However, the sharp phonon bands characteristic of the crystal are absent and we estimate that the contamination with (nano) crystals is less than 2% (see S4). Crucially, the Raman map of the phonon region (Fig. 3a) shows no significant anisotropy.

After a cold-crystallisation cycle, the low-frequency Raman spectrum of the droplet shows the development of strong phonon bands at 58, 105, and 120 cm^−1^, characteristic of the crystalline phase. The Raman map of the phonon region is now highly anisotropic, demonstrating that the (nano) crystallites that have apparently formed are aligned with (what was) the LC director. A series of temperature-dependent kinetics experiments on the rate of growth of the phonon bands during cold crystallisation, show the formation of the polycrystalline phase to be an activated process with an activation energy of approximately 90 kJ/mol.

**Figure 4:**
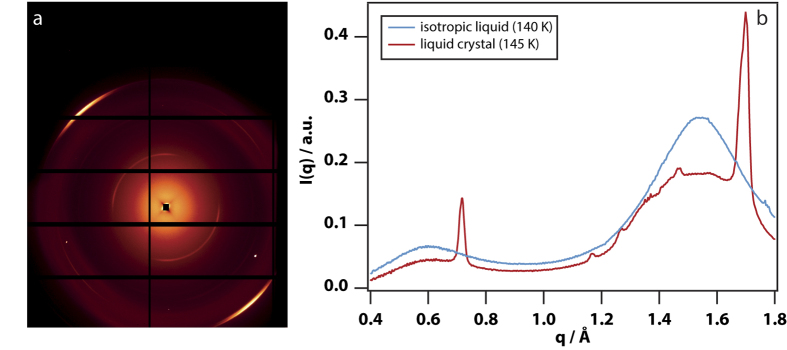
Microfocus wide-angle x-ray scattering (WAXS) data of *n*-butanol at 145 K proving that the new phase is an LC phase. **(a)** A WAXS diffraction pattern taken in an area with a diameter of 10 μm approximately to the left of the centre of a 400-μm diameter LC droplet. The intense diffuse bands at the left top and right bottom correspond with a scattering vector q = 1.7 Å^−1^. **(b)** WAXS scattering intensity in the range 0.4 to 1.8 Å^−1^ for isotropic liquid at 140 K and LC at 145 K (corresponding to the data in (**a**)).

Wide-angle x-ray scattering (WAXS) experiments were carried out using a 10-μm diameter microfocus beam at the Diamond Light Source. This was used to examine the supra-molecular structure inside an LC droplet when it had grown at 140 K to a diameter of a few hundred micrometres. WAXS of isotropic liquid at 140 K (see Fig. 4) shows two diffuse bands at *q* = 0.6 and 1.55 Å^−1^ as observed previously^16^,^18^. When the x-ray beam is focused off-centre in an LC droplet, additional less-diffuse anisotropically scattered bands can be seen at *q* = 0.718 Å^−1^ and *q* = 1.7 Å^−1^ (Fig. 4), consistent with an LC phase. The WAXS data also show weak crystal peaks consistent with less than 1.8% contamination with the crystalline phase (see S4). The orientation of the anisotropic scattering rotates as the microfocus beam is scanned around the droplet centre, consistent with the LC director pointing away from the centre. The less diffuse bands have widths of 0.017 and 0.037 Å^−1^ respectively, corresponding to a coherent domain size of about 90 molecules.

## Discussion

Polarisation microscopy strongly suggests that the newly nucleated phase is a spherically symmetric LC. Unusually, this transition in *n*-butanol can be significantly supercooled. However, polarisation microscopy cannot rule out the possibility that the droplets are spherulites^22^, that is, spherical polycrystalline growth patterns consisting of microcrystals unresolvable by light microscopy.

**Figure 5:**
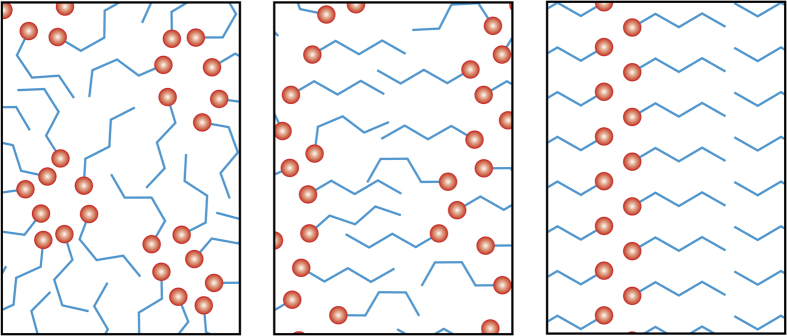
Cartoon of the molecular structure in n-butanol showing how the LC order frustrates the formation of the crystal. (left) The molecular structure of the isotropic liquid, where a red sphere represents the OH-group. (middle) The rippled lamellar LC arrangement compatible with the data taken in the LC droplets. (right) The known structure of the crystal.

The Raman spectrum of isotropic liquid in the OH-stretch region is very broad corresponding to the formation of 1 to 3 hydrogen bonds^23^ and broadening caused by orientational disorder. Based on computer simulations of *n*-octanol^24^, small-angle x-ray scattering studies of *n*-alcohols^25^, and dielectric-relaxation studies^26^, it can be concluded that the isotropic liquid forms clusters with a polar hydrogen-bonded core and a nonpolar shell consisting of overlapping alkyl chains (see Fig. 5). After the liquid–LC transition, the OH-stretch bands blue shift and narrow, becoming very similar to the spectrum of the crystal. This shows that in both the crystal and LC, only two hydrogen bonds are present (one donated and one accepted) and orientational disorder is strongly reduced.

Based on the OH-stretch Raman-scattering intensity around 3100 cm^−1^, we estimate (see S1) that less than 2.8% of isotropic liquid remains, showing the phase transition is complete within the accuracy of the experiment. The intensity of the main OH-stretch bands is highly anisotropic in the droplet before and after cold crystallisation, as well as in the spherulitic crystal growth outside the droplet. In crystalline *n*-butanol, the hydrogen-bond network forms infinite chains in the [100] direction^16^ causing the OH bonds to be aligned giving rise to a large anisotropy. If the newly formed phase is indeed an LC, then the observed anisotropy is consistent with the OH bond being aligned parallel with the LC director. However, if the new phase is not an LC but a spherulite, then the anisotropy implies a complete conversion to a spherulitic polycrystalline phase.

In the low-frequency Raman spectrum of any crystalline phase, one expects to observe sharp peaks due to phonon modes. Indeed, the spherulitic growth around the droplet that is induced by a cold-crystallisation cycle, displays strong phonon bands at 58 and 72 cm^−1^. However, in the droplet these peaks are very weak before cold crystallisation, leading us to estimate (see S4) a less than 2% contamination with crystals. After cold crystallisation the phonon peaks in the droplet are identical to those in the crystal. This is consistent with analysis of WAXS data, which leads to an estimate (see S4) of less than 1.8% contamination with crystals. These results are inconsistent with a complete conversion to a spherulitic crystalline phase and inconsistent with an arrested crystallisation scenario. However, these results are consistent with a full conversion to an LC that is contaminated with less than 2% (nano) crystallites. It also explains the lack of significant anisotropy in the phonon region in the LC.

Kinetic measurements during cold crystallisation cycles are consistent with a conversion of the LC to an oriented (spherulitic) polycrystalline phase through an activated process. The OH-stretch bands narrow to that seen in the crystal; phonon peaks grow to a size seen in the crystal; and the Raman intensity in the low-frequency region becomes highly anisotropic. This proves that the LC phase is metastable and frustrates crystallisation on lowering the temperature.

In the WAXS data, anisotropic diffuse diffraction bands are seen demonstrating the presence of an LC phase. The diffraction peak at *q* = 0.718 Å^−1^ correlates with the (001) scattering peak in the crystal and a Bragg separation of 8.75 Å. In the crystal, this distance corresponds with the distance between planes in which the *n*-butanol molecules hydrogen bond^16^. The diffraction peak at *q* = 1.7 Å^−1^ correlates with (012) scattering peak in the crystal and a Bragg separation of 3.7 Å. The (012) reflection corresponds with nearest neighbour packing perpendicular to the long axis of *n*-butanol. Therefore, the WAXS data are inconsistent with a nematic phase, inconsistent with growth of a spherulitic crystalline phase, but consistent with a rippled lamellar LC or gel phase. Raman spectra in the CH-stretch region (see Figure S8) show^27^ a mixture of gauche and trans conformations of the alkyl chains in the LC.

Thus, we have observed a transition from an isotropic liquid to an LC phase in *n*-butanol taking place entirely in the supercooled region, not a polyamorphic liquid-liquid transition between two isotropic phases as suggested earlier^8^. It also does not involve the formation of a mixed phase of (nano) crystallites embedded in untransformed liquid or an “arrested crystallisation”^13^,^14^,^15^,^16^,^18^. The liquid–LC transition is estimated to start at 150 K and can be carried out to temperatures as low as 120 K, just above the isotropic-liquid glass-transition temperature^14^, corresponding to a remarkable 30 K of supercooling. This large degree of supercooling is not normally seen in liquid–LC transitions. Because of the large degree of supercooling, the LC phase is likely to be very viscous leading to the sharp interfaces seen in Fig. 1(e).

On warming towards the melting temperature, the isotropic liquid cold crystallises but the crystals do not penetrate the LC droplet (see supplementary video and Figure S9). This again shows that the LC phase is metastable and frustrating the formation of the crystal consistent with the kinetic measurements. These behaviours are inconsistent with the LC being an Ostwald step-rule intermediate on the crystallisation pathway between the isotropic liquid and the crystal. In the crystal, the molecules are stacked anti-parallel forming infinite chains of hydrogen bonds^16^. We find that in the LC the hydrogen-bonded chain is disordered (rippled) and that the alkane tails have a distribution of gauche and trans conformations. (see Fig. 5).

The Raman spectra show that the liquid–LC transition involves the severing of the same approximate number of hydrogen bonds as in crystallisation due to the breakup of hydrogen-bonded clusters present in the isotropic liquid. Therefore, the interfacial energy will be approximately equally large for both transitions. This explains the unusually large degree of supercooling that can be achieved. It also explains why the LC phase forms droplets rather than schlieren textures.

At high enough temperatures, the molecules have sufficient kinetic energy to escape from the frustrated LC state and evolve towards thermodynamic equilibrium. This mechanism gives rise to mixtures of micro-crystallites and untransformed liquid, and explains much of the long-standing confusion in the literature^13^,^14^,^15^,^18^. Studies on *n*-butanol using Brillouin scattering found crystalline-like acoustic phonon peaks in the new phase inconsistent with those observed in the crystal^14^,^15^. This result can now be understood as coming from the partial ordering of the LC. The recently reported disappearance of the Debye process combined with the survival of the liquid-like β-process^26^ during the transition is similarly fully consistent with the breakup of the hydrogen-bonded clusters in the isotropic liquid and formation of the LC.

The formation of the LC phase from the isotropic liquid is fully consistent with existing calorimetric measurements^8^,^14^,^15^,^28^. In particular, calorimetric measurements, starting from the *n*-butanol glass, using different heating speeds^15^ show an exothermic peak corresponding to the “glacial phase” and a separate exothermic first-order transition into the stable crystal above 155 K. Thus, the “glacial phase” can now be reinterpreted as the slow formation of a rippled lamellar LC phase.

The idea of geometric frustration as a mechanism to explain supercooling and glass formation was introduced 60 years ago^29^ and has been widely applied to magnetic systems^30^, and colloid, polymer, and granular materials^31^,^32^. The results presented here demonstrate a form of frustration pertinent to molecular liquids in which local interactions give rise to partially ordered states (an LC in this case) that are incompatible with the (thermodynamically more stable) crystalline state, giving rise to frustration.

## Materials and Methods

### Materials

Experiments were carried out on anhydrous *n*-butanol (99.95% pure, 0.001% water content as determined by Karl Fischer titration) purchased from Sigma-Aldrich and used as supplied. For all microscopy experiments, a sample thickness of 11.58 ± 0.19 μm was used, controlled by glass monodisperse particle standards (Whitehouse Scientific). Temperature was controlled to ± 0.1 K using a Linkam THMS600 microscope stage. In the experiments, the samples were quenched and held at a selected temperature^9^.

### Microscopy

Polarisation microscopy images and videos were recorded using a Nikon Eclipse 50i microscope^33^. Confocal Raman microscopy experiments were performed using a Horiba LabRAM HR confocal microscope system. The excitation source was a vertically polarised 28-mW frequency-doubled DPSS laser operating at 532 nm^33^. Detailed Raman spectra were collected by averaging 8 scans of 32 s each (4 minutes acquisition time per spectrum).

Raman maps were acquired using the LabRAM confocal Raman system. The samples were prepared by quenching from room temperature to 140 K and waiting for approximately 20 minutes for a suitable LC droplet to grow. A typical droplet formed under these circumstances had a diameter of ~50 μm. At this point the sample could be rapidly quenched to 110 K (below the glass transition temperature of the isotropic liquid) to arrest all growth and to allow leisurely collection of the Raman data. Alternatively, the sample temperature could be raised rapidly to 173 K (10 K below the melting temperature of *n*-butanol) to induce cold crystallisation of the isotropic liquid followed by a rapid quench to 110 K as before.

The images shown in Fig. 3 have a 1 μm × 1 μm pixel size and the Raman spectrum was acquired in each pixel by averaging for ~0.5 s at full laser power. The OH-stretch maps were prepared by determining the amplitude in the 3285–3305 cm^−1^ and 3370–3390 cm^−1^ ranges after subtracting a baseline value determined from the amplitude in the 3050–3090 cm^−1^ range. The phonon maps were similarly prepared by subtracting the baseline from the 20–35 cm^−1^ range to get the amplitude in the 55–60 cm^−1^ and 115–130 cm^−1^ ranges.

### X-ray diffraction

WAXS experiments were carried out at beamline I22 in the Diamond Light Source using a microfocus setup. The x-rays had a photon energy of 14 keV (wavelength *λ* = 0.89 Å), were focused to a spot with a diameter of 10 μm, and were detected with a Pilatus 2 M (Dectris) detector placed 36 cm behind the sample using an acquisition time of 10 seconds. The scattering wave-vector resolution of this setup is estimated at 0.003 Å^−1^. Data were taken over a scattering vector range 0.1–3 Å^−1^. Samples with a 0.5-mm thickness were held between 25-μm mica windows in a Linkam DSC600 cryogenic stage. The WAXS scattering intensity data shown here had a diffuse background from mica subtracted by assuming a negligible WAXS intensity at 0.3 Å^−1^.

### Data availability

The microscopy images, Raman spectra, and x-ray data used in this study are available in the Enlighten Research Data Repository (University of Glasgow) with the identifier doi: 10.5525/gla.researchdata.382.

## Additional Information

**How to cite this article**: Syme, C. D. *et al*. Frustration of crystallisation by a liquid–crystal phase. *Sci. Rep.* **7**, 42439; doi: 10.1038/srep42439 (2017).

**Publisher's note:** Springer Nature remains neutral with regard to jurisdictional claims in published maps and institutional affiliations.

## Supplementary Material

Supplementary Information

Supplementary video

## References

[b1] Reinitzer F. Beitrage zur Kenntniss des Gholesterins. Monatsh. Chem. 9, 421–441 (1888).

[b2] Blinov L. M. Structure and Properties of Liquid Crystals. (Springer Science and Business Media, 2010).

[b3] Armitage D. & Price F. P. Supercooling and Nucleation in Liquid Crystals. Mol. Cryst. Liq. Cryst. 44, 33–44 (1978).

[b4] Bhat M. H. . Vitrification of a monatomic metallic liquid. Nature 448, 787–790 (2007). 17700696 10.1038/nature06044

[b5] Mcmillan P., Wilson M., Daisenberger D. & Machon D. A density-driven phase transition between semiconducting and metallic polyamorphs of silicon. Nat Mater 4, 680–684 (2005). 16113681 10.1038/nmat1458

[b6] Katayama Y. . A first-order liquid-liquid phase transition in phosphorus. Nature 403, 170–173 (2000). 10646596 10.1038/35003143

[b7] Kurita R. & Tanaka H. Critical-like phenomena associated with liquid-liquid transition in a molecular liquid. Science 306, 845–848 (2004). 15514150 10.1126/science.1103073

[b8] Kurita R. & Tanaka H. On the abundance and general nature of the liquid-liquid phase transition in molecular systems. J Phys-Condens Mat 17, L293–L302 (2005).

[b9] Mosses J., Syme C. D. & Wynne K. Order Parameter of the Liquid–Liquid Transition in a Molecular Liquid. J Phys Chem Lett 6, 38–43 (2015). 26263088 10.1021/jz5022763

[b10] Murata K.-I. & Tanaka H. Microscopic identification of the order parameter governing liquid–liquid transition in a molecular liquid. Proc Natl Acad Sci USA 112, 5956–5961 (2015). 25918385 10.1073/pnas.1501149112 PMC4434750

[b11] Kurita R., Murata K.-I. & Tanaka H. Control of fluidity and miscibility of a binary liquid mixture by the liquid-liquid transition. Nat Mater 7, 647–652 (2008). 18604215 10.1038/nmat2225

[b12] Dressel C., Reppe T., Prehm M., Brautzsch M. & Tschierske C. Chiral self-sorting and amplification in isotropic liquids of achiral molecules. Nat Chem 6, 971–977 (2014). 25343601 10.1038/nchem.2039

[b13] Wypych A., Guinet Y. & Hedoux A. Isothermal transformation of supercooled liquid n-butanol near the glass transition: Polyamorphic transitions in molecular liquids investigated using Raman scattering. Phys Rev B 76, 144202 (2007).

[b14] Hassaine M. . Thermal properties and Brillouin-scattering study of glass, crystal, and &quot;glacial&quot; states in n-butanol. J Chem Phys 131, 174508 (2009). 19895026 10.1063/1.3258645

[b15] Shmytko I. M., Jimenez-Rioboo R. J., Hassaine M. & Ramos M. A. Structural and thermodynamic studies of n-butanol. J Phys-Condens Mat 22, 195102 (2010). 10.1088/0953-8984/22/19/195102 21386446

[b16] Derollez P., Hedoux A., Guinet Y., Danede F. & Paccou L. Structure determination of the crystalline phase of n-butanol by powder X-ray diffraction and study of intermolecular associations by Raman spectroscopy. Acta Crystallogr B 69, 195–202 (2013). 10.1107/S2052519213004843 23719706

[b17] Bolshakov B. V. & Dzhonson A. G. On the number of amorphous phases in n-butanol. Doklady Phys Chem 393, 318–320 (2003).

[b18] Hedoux A., Guinet Y., Paccou L., Derollez P. & Danede F. Vibrational and structural properties of amorphous n-butanol: A complementary Raman spectroscopy and X-ray diffraction study. J Chem Phys 138, 214506 (2013). 23758387 10.1063/1.4808159

[b19] Hunt N. T., Turner A. R. & Wynne K. Inter- and intramolecular hydrogen bonding in phenol derivatives: a model system for poly-L-tyrosine. J Phys Chem B 109, 19008–19017 (2005). 16853447 10.1021/jp052964o

[b20] Fecko C., Eaves J. & Tokmakoff A. Isotropic and anisotropic Raman scattering from molecular liquids measured by spatially masked optical Kerr effect spectroscopy. J Chem Phys 117, 1139–1154 (2002).

[b21] Fukasawa T. . Relation between dielectric and low-frequency Raman spectra of hydrogen-bond liquids. Phys Rev Lett 95, 197802 (2005). 16384025 10.1103/PhysRevLett.95.197802

[b22] Gránásy L., Pusztai T., Tegze G., Warren J. & Douglas J. Growth and form of spherulites. Phys Rev E 72, 011605 (2005). 10.1103/PhysRevE.72.011605 16089977

[b23] Paolantoni M., Sassi P., Morresi A. & Cataliotti R. S. Infrared study of 1-octanol liquid structure. Chem Phys 310, 169–178 (2005).

[b24] Maccallum J. L. & Tieleman D. P. Structures of Neat and Hydrated 1-Octanol from Computer Simulations. J Am Chem Soc 124, 15085–15093 (2002). 12475354 10.1021/ja027422o

[b25] Triolo A., Russina O., Fazio B., Triolo R. & Di Cola E. Morphology of 1-alkyl-3-methylimidazolium hexafluorophosphate room temperature ionic liquids. Chem Phys Lett 457, 362–365 (2008).

[b26] Jensen M. H., Alba-Simionesco C., Niss K. & Hecksher T. A systematic study of the isothermal crystallization of the mono-alcohol n-butanol monitored by dielectric spectroscopy. J Chem Phys 143, 134501–134509 (2015). 26450317 10.1063/1.4931807

[b27] Chen L. . Identification of Alcohol Conformers by Raman Spectra in the C–H Stretching Region. J Phys Chem A 119, 3209–3217 (2015). 25774682 10.1021/jp513027r

[b28] Hassaine M. & Ramos M. A. Calorimetric studies at low temperatures of glass-forming 1-butanol and 2-butanol. Phys. Status Solidi A 208, 2245–2248 (2011).

[b29] Frank F. C. Supercooling of liquids. Proc R Soc Lon Ser-A 215, 43–46 (1952).

[b30] Ramirez A. Strongly geometricall frustrated magnets. Ann. Rev. Mat. Sci. 24, 453–480 (1994).

[b31] Nowak E. R., Knight J. B., Ben-Naim E., Jaeger H. M. & Nagel S. R. Density fluctuations in vibrated granular materials. Phys Rev E 57, 1971–1982 (1998).

[b32] Han Y. . Geometric frustration in buckled colloidal monolayers. Nature 456, 898–903 (2008). 19092926 10.1038/nature07595

[b33] Mosses J., Turton D. A., Lue L., Sefcik J. & Wynne K. Crystal templating through liquid-liquid phase separation. Chem Commun 51, 1139–1142 (2015). 10.1039/c4cc07880b 25466237

