# Supplementary material for: Frustration of crystallisation by a liquid–crystal phase
Source: Sci Rep. 2017 Feb 17;7:42439. doi: 10.1038/srep42439 (PMC5314399; doi:10.1038/srep42439)
Supplement: Supplementary Information [file srep42439-s1.pdf]

Supporting Information
Frustration of crystallisation by a liquid–crystal phase

Christopher D. Syme,[a] Joanna Mosses,[a] Mario González Jiménez,[a] Olga Shebanova,[b] Finlay Walton,[a] and Klaas Wynne[a,*]
[a] School of Chemistry, WestCHEM, University of Glasgow, UK
[b] Diamond Light Source, Harwell Science and Innovation Campus, Oxfordshire, UK

S1 – Experimental determination of contamination of the LC with isotropic liquid

One of the most important questions to address is whether the transformation described in the accompanying manuscript is caused by either a liquid–LC transition or by an aborted crystallisation. If the latter were the case then one should observe contributions from the isotropic liquid in the Raman spectra of the new phase.

Figure S1 shows the Raman spectra of the various phases (isotropic liquid, LC made by quenching to 140 K, and crystal) in the region between the CH-stretch and OH-stretch bands all taken at the same temperature (140 K). The falling intensity in the Raman spectrum of the crystal in the range 3000-3150 cm^{-1} is caused by the tail of the CH-stretch band. In this frequency region, the crystal spectrum can be considered a "background" with any Raman intensity above this background caused by the OH-stretch in either the isotropic liquid or the LC. The Raman spectrum of the LC is more intense than that of the crystal between 3100 and 3290 cm^{-1} because the OH-stretch bands are broadened compared to the crystal spectrum. The OH-stretch band of the isotropic liquid can clearly be observed above 3050 cm^{-1} and can therefore be used to estimate the fraction of isotropic liquid left after the transformation to the new phase. In the range 3080-3110 cm^{-1} (shown as a grey band in Figure S1), the Raman intensity in the LC spectrum has fallen to the background (the crystal spectrum). However, in the same range, the Raman intensity in the isotropic-liquid spectrum is well above the background. If any isotropic liquid was left in the new phase, one should observe above-background intensity in the 3080-3110 cm^{-1} range. Thus, using the data in this range, we calculate a **mean contribution of the isotropic liquid to the LC spectrum of less than 2.8%.** Estimates obtained from bands at 510, 650, 750, 850, 1032, and 2720 cm^{-1} are all less than 5-10% consistent with the estimate obtained from the OH-stretch region.

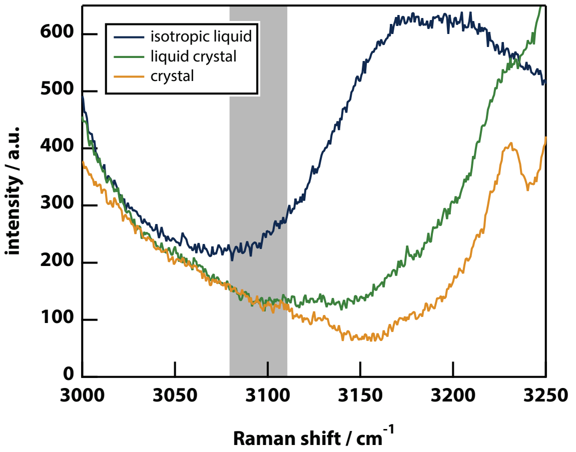

Figure S1 ***Raman spectra in the OH-stretch region used to determine the degree to which isotropic liquid is present in the LC droplets***. *The Raman spectra of n-butanol taken before transformation (isotropic liquid), after the phase transition (LC), and after cold crystallisation (crystal) at 140 K. The grey bar shows the range 3080-3110 cm^{-1} used to estimate how much isotropic liquid is left in the LC droplet.*

S2 – Determination of the anisotropy in the Raman scattering intensity

In this study, Raman maps were made of the LC droplets and their immediate surrounding before and after cold crystallisation (see Figure S2) by using a vertically polarised excitation laser and un-polarised detection of the Raman scattered light. As the LC director is pointing away from the droplet centre, the projection of the laser polarisation vector onto the molecular axis varies throughout the droplet. This gives rise to anisotropic Raman maps as can be seen in Figure S3. Depending on the molecular Raman polarisability tensor of a particular vibrational mode, these maps are more or less intense in directions parallel or perpendicular to the excitation laser polarisation.

To quantify the degree of anisotropy, the Raman intensity is measured at two points displaced along the x- and y-axis. In Figure S3, we have indicated two typical sample positions with crosses where the measured Raman intensities are I_x and I_y respectively. The anisotropy is defined here as I_y/I_x.

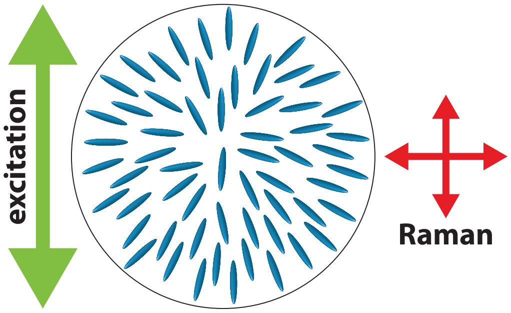

Figure S2 ***Diagram showing the experimental determination of the anisotropy in Raman intensity***. *The droplet (centre) contains a LC phase in which the director is pointing away from the droplet centre. Raman mapping experiments are carried out using a vertically polarised excitation laser while Raman scattered light is detected without polarisation selection.*

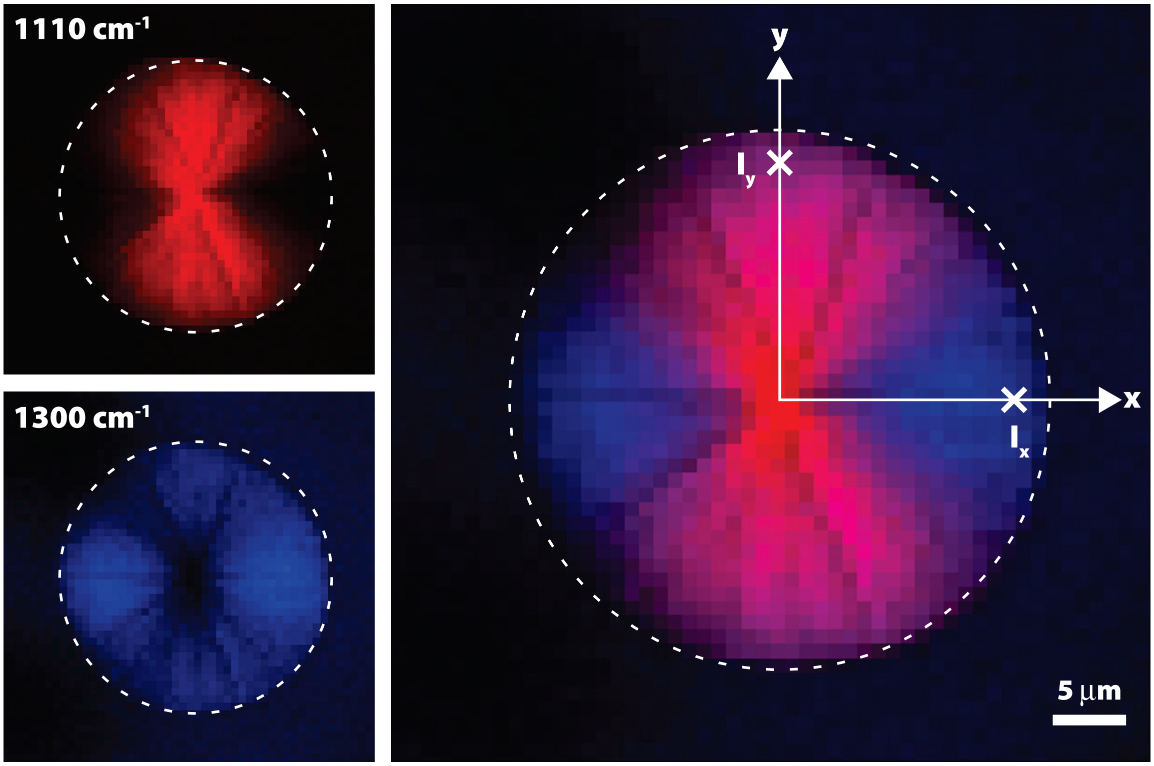

Figure S3 ***Determination of anisotropy from Raman maps***. *Shown are Raman maps of a LC droplet in n-butanol formed at 140 K and measured at 110 K. On the left are Raman maps of vibrational resonances at 1110 and 1300 cm^{-1}, while on the right these are combined in a single map. Also shown are two typical points displaced along the x and y-axis that are used in the calculation of the anisotropy.*

S3 – Raman polarisability tensor and anisotropy

The derivative of the Raman polarisability tensor ($d\alpha / dq$) of the OH-stretch vibrational mode was calculated for ethanol (which is faster to calculate than n-butanol but should give the same result for the OH-stretch) using the Gaussian 09 software package[1] using B3LYP/6-31+G(d,p) level of theory. The polarisability tensor is dominated by the polarisability in the direction of the O-H bond with a small rotation in the direction of the C-O bond. The eigenvalues of the polarisability tensor are -2.90, -0.28, and -0.12 Å^2. Using these eigenvalues, it is straightforward to calculate the strength of the OH-stretch Raman band as a function of the direction of the electric field vector.

The unit cell of the crystalline form of *n*-butanol contains two molecules. Using the known coordinates of the OH groups,[2] one can calculate the Raman scattering strength as function of the direction of the electric field vector within the crystal lattice. The maximum ratio of the strongest *vs*. the weakest Raman scattering strength calculated this way is 7.6. When *n*-butanol (liquid 1) is cold crystallised, it forms a polycrystalline sample with randomly oriented but easily discernible crystals. Raman microscopy measurements on the OH-stretch mode of such a polycrystalline sample reveals a maximum intensity ratio of 5-10, consistent with the theoretical value.

S4 – Experimental determination of contamination of the LC with (nano) crystals

In order to estimate the crystalline component in the LC droplets prior to cold crystallisation, spectra were taken from LC droplets both before and after cold crystallisation and the peak intensities were compared in the phonon region, specifically at 58 cm^{-1} where a prominent peak appears upon cold crystallisation and at ~71-72 cm^{-1}. The shape of the Raman spectrum of the droplets after cold crystallisation is identical (within the signal to noise ratio) to that of poly-crystalline *n*-butanol obtained by cold crystallising the isotropic liquid.

It was necessary to subtract a background caused by librations and hydrogen-bond modes[3,4] from all data. The Raman bands corresponding to these underdamped molecular motions are not expected to change very much between liquid, LC, and crystal. Therefore, the contribution from librations and hydrogen-bond modes was estimated by using the isotropic liquid spectrum measured in the same setup.

Figure S4 (a) and (b) show the Raman spectra of a droplet before and after cold crystallisation with the librations and hydrogen-bond mode background for both traces shown in grey. The resulting background subtracted spectra are shown in Figure S4 (c). The intensity of the background-subtracted data below 80 cm^{-1} is interpreted as being due to phonon modes of crystalline butanol. From these traces, we estimate that the amount of crystal contamination in the LC droplet prior to cold crystallisation is **less than 2%** based on the intensity of the 58-cm^{-1} phonon band and below 18% based on the intensity of the 72-cm^{-1} phonon band. This implies that the 72-cm^{-1} phonon band is sensitive to a degree of order that remains on melting the crystal to form the LC.

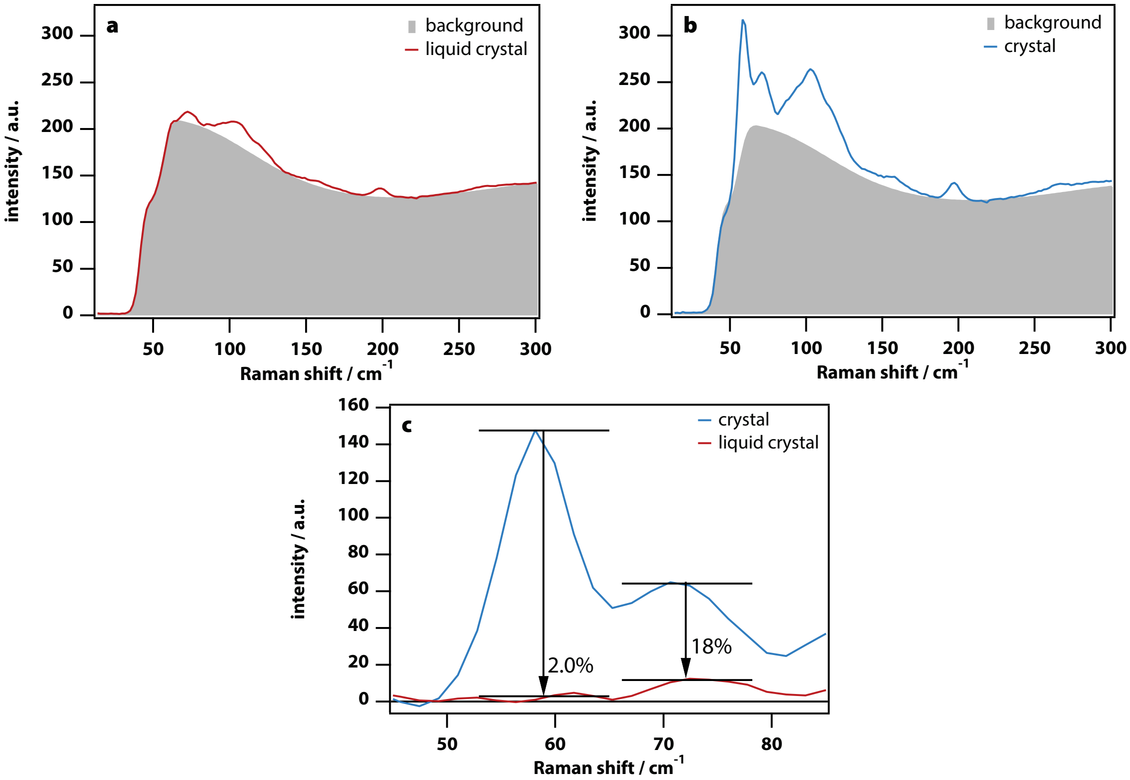

Figure S4 ***Low-frequency Raman spectra to determine the degree to which crystals are present in the LC droplets before cold crystallisation***. *The low-frequency Raman spectra of n-butanol taken after the liquid–LC transformation and in the same location after cold crystallisation. (**a**) The Raman spectrum of LC with the Raman spectrum of the isotropic liquid shown in grey. (**b**) Idem after a cold-crystallisation cycle. (**c**) The same spectra as shown in (a) and (b) with the isotropic-liquid spectrum subtracted.*

Another way to estimate the crystalline component in the LC droplets prior to cold crystallisation involves using wide-angle x-ray scattering (WAXS). Figure S5 shows the microfocus WAXS data collected on a LC droplet before cold-crystallisation and a polycrystalline sample. The polycrystalline WAXS data shows numerous prominent sharp

peaks. Some of these, such as the (001) and (012) peaks, re-appear in the LC WAXS data although much broadened as expected. Other peaks, such as the (101) peak, are essentially absent in the LC and can be used to estimate the degree of contamination with crystals. Based on the reduction of the intensity of peaks such as the (101) peak, it can be estimated that the contamination of the LC phase with crystals is **less than 1.8%**.

Thus, two independent techniques come to identical conclusions (within the signal to noise ratio) that **the LC phase contains no more than 2% crystalline material**.

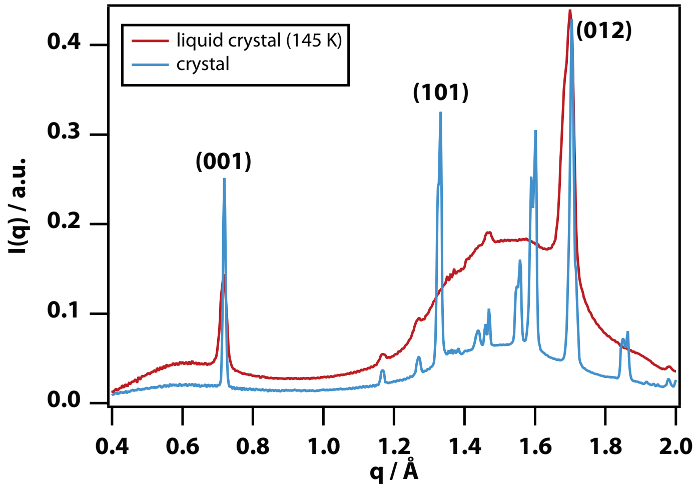

Figure S5 *Wide-angle x-ray scattering (WAXS) data of LC and poly-crystalline n-butanol at 145 K in the range 0.4 to 2.0 Å^{-1} showing the broad diffraction peaks in isotropic liquid that demonstrate the presence of LC order.*

S5 – Supplementary video

This video shows the formation of LC droplets in n-butanol at 140 K, followed by cold crystallisation at 173 K, and melting at 185 K. The crystals that grow at 173 K do not penetrate the LC droplet.

S6 – Supplementary figures

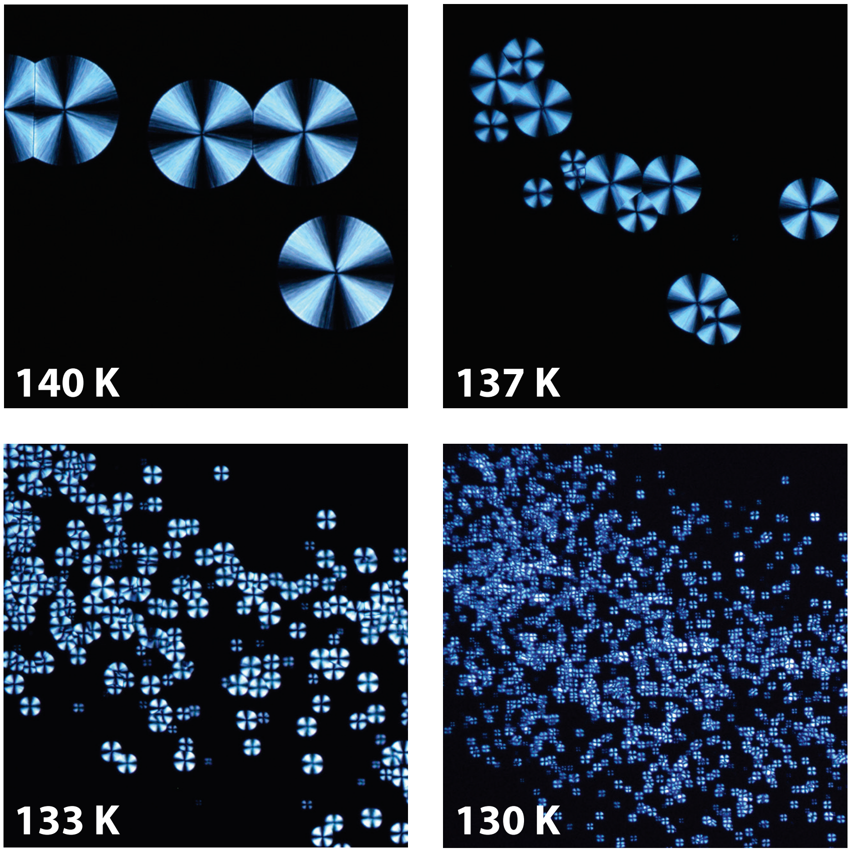

Figure S6 ***Polarisation microscopy of the LC phase as a function of temperature***. *All images were taken 30 minutes after quenching to a temperature of 130, 133, 137, and 140 K respectively.*

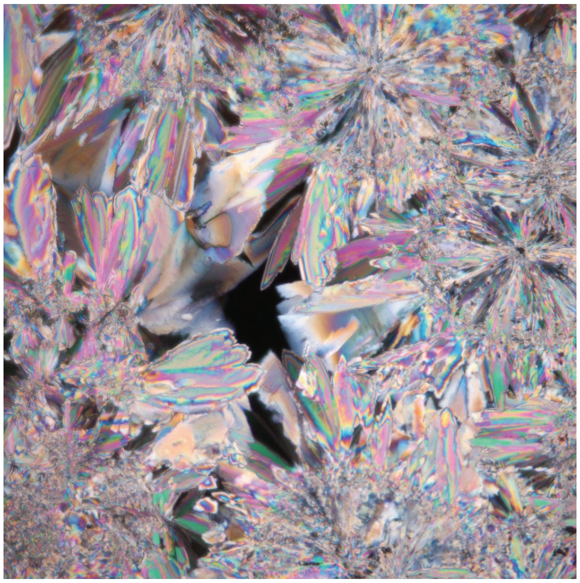

Figure S7 ***Polarisation microscopy of the polycrystalline phase of n-butanol***. *The polycrystalline phase is obtained by cold crystallisation at mild (~10 K) supercooling.*

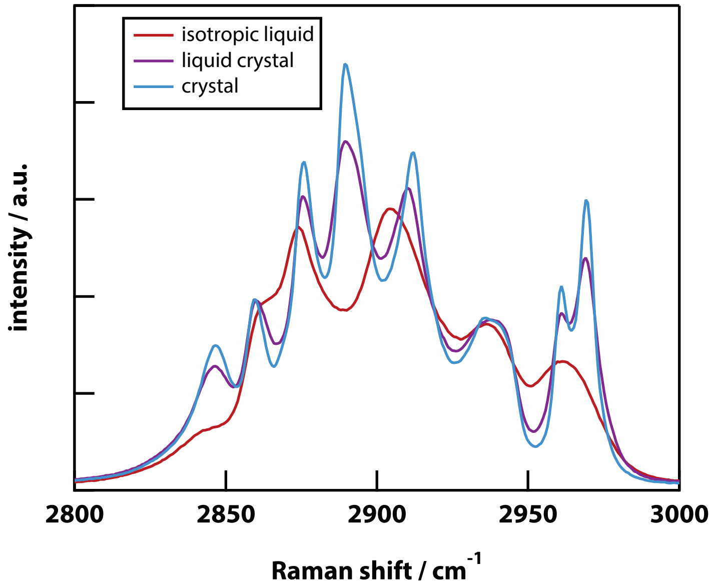

Figure S8 Raman spectra in the CH-stretch region. The Raman spectra of *n*-butanol taken before transformation (isotropic liquid), after the phase transition (LC), and after cold crystallisation (crystal) at 140 K.

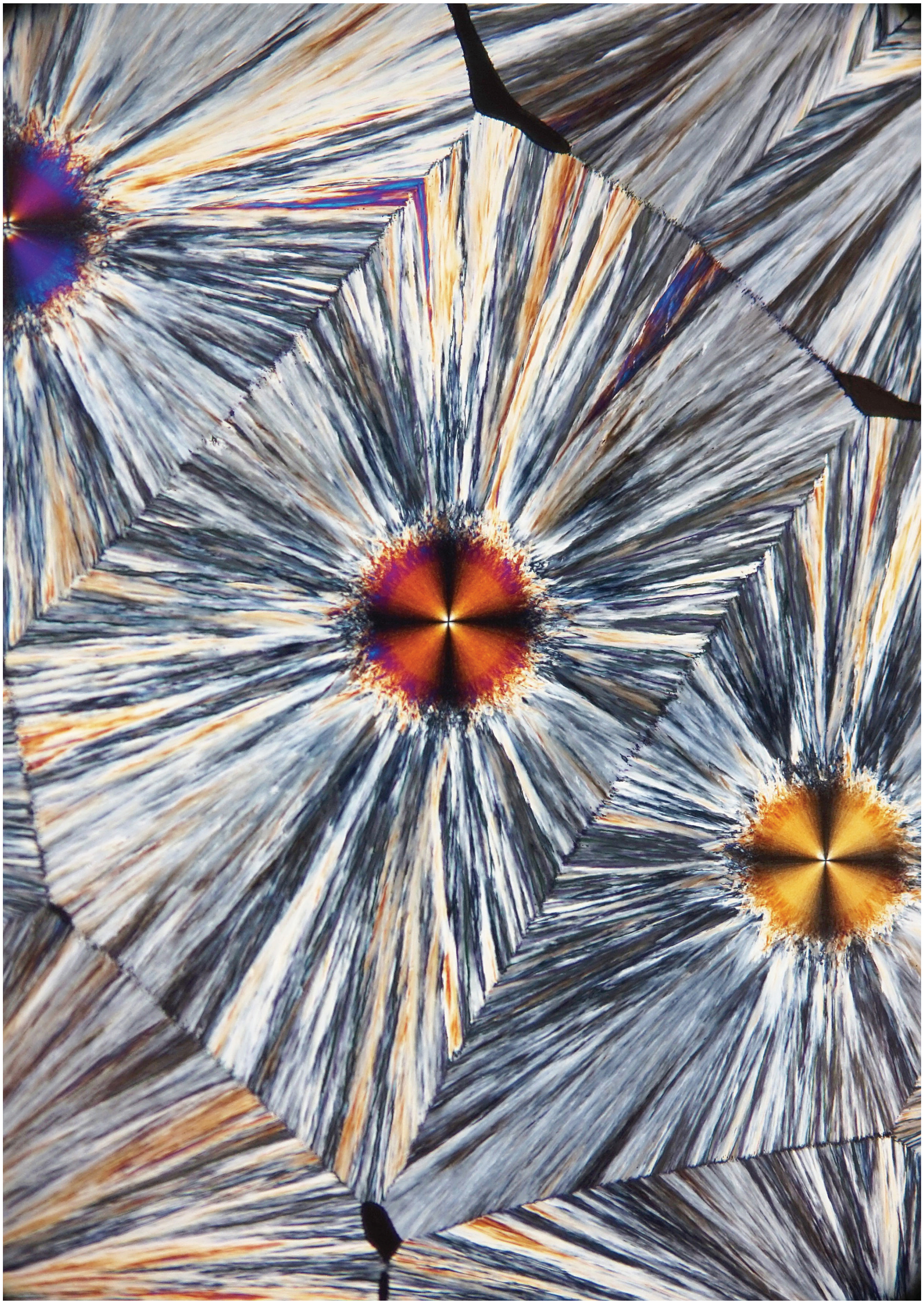

Figure S9 ***Polarisation microscopy photo of LC droplets encased in the poly-crystalline phase showing that the LC droplets resist crystallisation***. *When n-butanol is supercooled (to 140 K in this case), a transition takes place to a LC phase that nucleates as droplets (the ~150 μm diameter brown and yellow droplets in the photo). When the remaining liquid is cold crystallised, it can be seen that the resultant crystals do not penetrate into the LC droplets, demonstrating that this phase frustrates the formation of the crystal. (See also the Supplementary video.)*

S7 – Supplementary references

1 Gaussian 09, Revision D.01, M. J. Frisch, G. W. Trucks, H. B. Schlegel, G. E. Scuseria, M. A. Robb, J. R. Cheeseman, G. Scalmani, V. Barone, B. Mennucci, G. A. Petersson, H. Nakatsuji, M. Caricato, X. Li, H. P. Hratchian, A. F. Izmaylov, J. Bloino, G. Zheng, J. L. Sonnenberg, M. Hada, M. Ehara, K. Toyota, R. Fukuda, J. Hasegawa, M. Ishida, T. Nakajima, Y. Honda, O. Kitao, H. Nakai, T. Vreven, J. A. Montgomery, Jr., J. E. Peralta, F. Ogliaro, M. Bearpark, J. J. Heyd, E. Brothers, K. N. Kudin, V. N. Staroverov, T. Keith, R. Kobayashi, J. Normand, K. Raghavachari, A. Rendell, J. C. Burant, S. S. Iyengar, J. Tomasi, M. Cossi, N. Rega, J. M. Millam, M. Klene, J. E. Knox, J. B. Cross, V. Bakken, C. Adamo, J. Jaramillo, R. Gomperts, R. E. Stratmann, O. Yazyev, A. J. Austin, R. Cammi, C. Pomelli, J. W. Ochterski, R. L. Martin, K. Morokuma, V. G. Zakrzewski, G. A. Voth, P. Salvador, J. J. Dannenberg, S. Dapprich, A. D. Daniels, O. Farkas, J. B. Foresman, J. V. Ortiz, J. Cioslowski, and D. J. Fox, Gaussian, Inc., Wallingford CT, 2013.

2 Derollez, P., Hedoux, A., Guinet, Y., Danede, F. & Paccou, L. Structure determination of the crystalline phase of n-butanol by powder X-ray diffraction and study of intermolecular associations by Raman spectroscopy. *Acta Cryst. B* **69**, 195-202 (2013).

3 Fecko, C., Eaves, J. & Tokmakoff, A. Isotropic and anisotropic Raman scattering from molecular liquids measured by spatially masked optical Kerr effect spectroscopy. *J Chem Phys* **117**, 1139-1154 (2002).

4 Fukasawa, T. *et al.* Relation between dielectric and low-frequency Raman spectra of hydrogen-bond liquids. *Phys Rev Lett* **95**, 197802 (2005).
